# Changes in N6-Methyladenosine Modification Modulate Diabetic Cardiomyopathy by Reducing Myocardial Fibrosis and Myocyte Hypertrophy

**DOI:** 10.3389/fcell.2021.702579

**Published:** 2021-07-21

**Authors:** Wenhao Ju, Kai Liu, Shengrong Ouyang, Zhuo Liu, Feng He, Jianxin Wu

**Affiliations:** ^1^Graduate School, Peking Union Medical College, Beijing, China; ^2^Department of Biochemistry and Immunology, Capital Institute of Pediatrics, Beijing, China; ^3^Beijing Municipal Key Laboratory of Child Development and Nutriomics, Beijing, China; ^4^Department of Biochemistry & Immunology, Capital Institute of Pediatrics-Peking University Teaching Hospital, Beijing, China; ^5^Beijing Tongren Hospital, Capital Medical University, Beijing, China

**Keywords:** diabetic cardiomyopathy, m^6^A, myocardial fibrosis, FTO, myocyte hypertrophy

## Abstract

In this study, we aimed to systematically profile global RNA N6-methyladenosine (m^6^A) modification patterns in a mouse model of diabetic cardiomyopathy (DCM). Patterns of m^6^A in DCM and normal hearts were analyzed via m^6^A-specific methylated RNA immunoprecipitation followed by high-throughput sequencing (MeRIP-seq) and RNA sequencing (RNA-seq). m^6^A-related mRNAs were validated by quantitative real-time PCR analysis of input and m^6^A immunoprecipitated RNA samples from DCM and normal hearts. A total of 973 new m^6^A peaks were detected in DCM samples and 984 differentially methylated sites were selected for further study, including 295 hypermethylated and 689 hypomethylated m^6^A sites (fold change (FC) > 1.5, *P* < 0.05). Gene ontology (GO) and Kyoto Encyclopedia of Genes and Genomes (KEGG) Pathway analyses indicated that unique m^6^A-modified transcripts in DCM were closely linked to cardiac fibrosis, myocardial hypertrophy, and myocardial energy metabolism. Total m^6^A levels were higher in DCM, while levels of the fat mass and obesity-associated (FTO) protein were downregulated. Overexpression of FTO in DCM model mice improved cardiac function by reducing myocardial fibrosis and myocyte hypertrophy. Overall, m^6^A modification patterns were altered in DCM, and modification of epitranscriptomic processes, such as m^6^A, is a potentially interesting therapeutic approach.

## Introduction

There are more than 450 million patients with diabetes worldwide, and by 2045 this number is predicted to increase to 693 million (Cho et al., 2018). Cardiovascular disease accounts for 50.3% of total deaths of patients with diabetes (Einarson et al., 2018). Diabetes not only increases the risk of heart failure, but also increases the mortality rate from heart failure by 2.5 times (Tan et al., 2020). Diabetic cardiomyopathy (DCM) is a metabolic cardiovascular disease resulting in decreased myocardial glucose consumption, modestly increased ketone metabolism, and significantly increased utilization of fatty acids (Ritchie and Abel, 2020; Tan et al., 2020). The main features of DCM are myocardial hypertrophy, cardiac fibrosis, coronary microvascular dysfunction, left ventricular enlargement, and weakened ventricular wall motion (Ritchie and Abel, 2020); however, the causal relationships among these complications are not clear. To date, therapies for DCM are limited and cannot prevent the eventual development of the disease. Therefore, additional treatment options are needed.

Although multiple aspects of epigenetic regulation, from DNA modification to protein modification, have been extensively studied in DCM, the role of RNA modification in the regulation of gene expression is just beginning to be elucidated (Gluckman et al., 2009; Zhang et al., 2018). The most pervasive internal mRNA modification is m^6^A methylation, which affects RNA metabolism throughout its life cycle (Nachtergaele and He, 2018). The m^6^A methyltransferase complex consists of at least three components, METTL3, METTL14, and WTAP, and m^6^A is catalyzed by a core writer complex, comprising the catalytic enzyme, METTL3, and is its allosteric activator, METTL14 (Wang P. et al., 2016; Wang X. et al., 2016). WTAP, a mammalian splicing factor, is an indispensable component of the m^6^A methyltransferase complex; it does not have methyltransferase activity, but can interact with METTL3 and METTL14 to influence cellular m^6^A deposition (Liu et al., 2014). In addition to this core complex, a writer complex, comprising VIRMA, RBM15 or RBM15B, and ZC3H3 subunits, has been identified (Patil et al., 2016; Wen et al., 2018; Yue et al., 2018). The m^6^A modification is dynamic and can be demethylated by FTO and ALKBH5 (Jia et al., 2011; Zheng et al., 2013; Mauer et al., 2017; Wei et al., 2018). FTO was the first m^6^A demethylase to be discovered (Jia et al., 2011) and it can demethylate internal m^6^A, cap m^6^Am, and tRNA m^1^A (Wei et al., 2018), whereas ALKBH5 specifically demethylates m^6^A by affecting RNA metabolism and mRNA export (Zheng et al., 2013). In addition, RNA-binding proteins that bind to m^6^A modification sites are regarded as m^6^A readers, which regulate the functions of m^6^A-modified RNAs through various mechanisms. YTH domain-containing proteins bind to RNA in an m^6^A-dependent manner (Li et al., 2014; Zhu et al., 2014). YTHDC1 is predominantly expressed in the nucleus, where it regulates mRNA splicing (Roundtree and He, 2016). YTHDC2 expresses in nuclear and cytosolic and promotes translation (Wojtas et al., 2017). The family of cytosolic YTHDF proteins includes YTHDF1, YTHDF2, and YTHDF3 (Du et al., 2016; Shi et al., 2017; Patil et al., 2018). YTHDF1 can facilitate the translation of m^6^A-modified mRNAs alongside translation initiation factors, YTHDF2 can cause degradation of m^6^A-containing RNAs, and YTHDF3 can facilitate both translation and degradation functions (Du et al., 2016; Shi et al., 2017; Patil et al., 2018). Besides the YTH domain-containing proteins, HNRNPA2B1, HNRNPC, HNRNPG, and IGF2BP1-3 have also been reported to preferentially bind to m^6^A-modified mRNAs (Alarcon et al., 2015a; Liu et al., 2015, 2017; Edupuganti et al., 2017; Huang et al., 2018).

The development of methods for analysis of genome-wide m^6^A topology has allowed extensive study of m^6^A-dependent regulation of RNA fate and function (Wang X. et al., 2014; Wang Y. et al., 2014; Alarcon et al., 2015b; Wang X. et al., 2015). Two independent studies reported m^6^A RNA methylomes in mammalian genomes for the first time using an m^6^A-specific methylated RNA immunoprecipitation approach, followed by high-throughput sequencing (MeRIP-seq) (Dominissini et al., 2012; Meyer et al., 2012). Emerging evidence indicates that m^6^A modification is associated with normal biological processes and with the initiation and progression of different types of heart disease (Dorn et al., 2019; Kmietczyk et al., 2019; Mathiyalagan et al., 2019; Mo et al., 2019; Song et al., 2019; Berulava et al., 2020; Gao et al., 2020; Kruger et al., 2020; Lin et al., 2020).

Dysregulation of m^6^A is associated with cardiac homeostasis and diseases, such as cardiac hypertrophy, cardiac remodeling, myocardial infarction, and heart failure (Dorn et al., 2019; Mathiyalagan et al., 2019; Mo et al., 2019; Song et al., 2019; Berulava et al., 2020; Gao et al., 2020; Lin et al., 2020); however, the transcriptome-wide distribution of m^6^A in DCM remains largely unknown. In this study, we report the m^6^A-methylation profiles of heart tissue samples from a db/db mice which is a well-established DCM model for diabetic complications and normal control mice (db/ +), and demonstrate highly diverse m^6^A-modified patterns in the two groups. We show that abnormal m^6^A RNA modifications in DCM likely modulate cardiac fibrosis, myocardial hypertrophy, and myocardial energy metabolism. Our results provide evidence that m^6^A modification is closely associated with DCM pathogenesis, and will facilitate further investigations of the potential targeting of m^6^A modification in DCM therapy.

## Materials and Methods

### Animals

This study was approved by the Ethics Committee of the Capital Institute of Pediatrics with the permit number: DWLL2019003. All procedures performed in the study complied with the relevant ethical standards. Leptin receptor-deficient (db/db) mice and control mice (db/ +) were purchased from Shanghai Model Organisms Center, which genetic background is C57BL/6J. All mice were used for experiments at 8–12 weeks old and were housed in constant 24 degrees cages with a 12 h alternating light/dark cycle and free access to water and food. To construct a diabetic heart disease model (DCM), mice were continuously fed to 24 weeks of age, then euthanized, hearts collected in 1.5 ml RNase-free centrifuge tubes, immediately immersed in liquid nitrogen to prevent RNA degradation, and finally stored at −80°C. Five pairs of db/db and db/ + heart samples were selected for RNA sequencing, and the remaining samples were saved for validation.

### Echocardiographic Assessment

Echocardiographic evaluation was blinded and conducted using a Vevo 2100 imaging system to the mouse at 24 weeks age, with two-dimensional guided M-mode image used to determine left ventricle size at papillary muscle level in the parasternal views (short axis and long axis), and calculate ejection fractions (EF) and fractional shortening (FS) by using standard equations (EF = (EDV-ES) × 100%/EDV; FS = (LVEDD-LVESD)/LVEDD × 100%). All measurements were averages from at least three beats.

### Western Blotting and Antibodies

Western blotting assays were conducted using standard protocols. In brief, mouse heart tissues were ground in a mortar and pestle under liquid nitrogen, then tissue lysate prepared using RIPA lysis buffer (Beyotime Biotechnology), supplemented with protease inhibitor. Blots were screened using specific antibodies: FTO (ab92821, 1:1,000, Abcam), GAPDH (5174, 1:10,000, CST), and m^6^A (ab208577, 1:500, Abcam).

### Histological Analysis

Mouse hearts were fixed in 4% paraformaldehyde for 12 h. After dehydration in 5% sucrose, samples were embedded in paraffin and hematoxylin and eosin (HE) staining performed on 5 μm thick sections.

In addition, frozen heart tissue samples were cut into 7 μm sections, incubated with wheat germ agglutinin (1:100) in the dark for 1 h, and then washed three times with PBS. DAPI (1:1000) was used to stain cell nuclei (10 min), followed by three washes with PBS. Water-soluble anti-fade mounting medium was dripped onto samples, which were then covered with glass cover slips and observed by confocal microscopy (Leica SP8). The cross-sectional areas of cardiomyocytes were calculated using Image-Pro Plus 6.0 software. To assess cardiac fibrosis, heart sections were stained using the standard Masson’s trichrome method.

### Overexpression of FTO

Empty adeno-associated virus (AAV-EV) and adeno-associated virus expressing FTO (AAV-FTO) under the control of a heart-specific *cTNT* promoter with an EGFP tag were constructed by Hanbio Biotechnology Ltd (Shanghai, China). The virus titer was approximately 1 × 10^12^ V.g/ml. At 16 weeks, db/db mice were injected with AAV-EV and AAV-FTO virus, respectively, via a tail vein (120 μl per mouse).

### m^6^A Dot Blot Assay

TRIzol (Invitrogen) was used to extract total RNA from mouse hearts. For mRNA denaturation, samples were heated at 95°C for 5 min, then immediately chilled on ice. Next, RNA samples were spotted on a positively charged nylon membrane (GE Healthcare) and cross-linked using an 80-degree hybridizer. Uncrosslinked RNA was eluted with PBS containing 0.01% Tween 20 for 5 min, then membranes incubated with anti-m^6^A antibody (1: 500 in PBS containing 0.01% Tween 20) at 4°C for 12 h after blocking with 5% skim milk (in PBS containing 0.01% Tween 20) for 1 h. Then, membranes were incubated with horseradish peroxidase-conjugated anti-rabbit IgG secondary antibody, gently agitated at room temperature for 1 h, and washed four times with PBS for 10 min, followed by development with chemiluminescence. Methylene blue staining was used to confirm that duplicate dots contained the same amount of total RNA.

### MeRIP-Seq

After five 24-week-old mice in each group were euthanized, total RNA samples were harvested from heart tissue specimens and quantified using a NanoDrop ND-1000 (Thermo Fisher Scientific, MA, United States). Then, complete mRNA was obtained by purification using Arraystar Seq-Star^TM^ ploy(A) mRNA Isolation Kit, and broken into fragments of approximately 100 nucleotides by incubation in fragmentation buffer [10 mM Zn^2+^ and 10 mM Tris–HCl (pH7.0)] at 94°C for 5–7 min. RNA fragments containing m^6^A methylation sites were enriched by immunoprecipitation using anti-m^6^A antibody (Synaptic Systems, 202003). A KAPA Stranded mRNA-seq Kit (Illumina) was used to construct sequencing libraries of post-enrichment m^6^A mRNA and input mRNA, which were then subjected to 150 bp paired-end sequencing on the Illumina NovaSeq 6000 platform.

FastQC (v0.11.7) was used for quality inspection of raw sequence data, and original sequence filtered using Trimmomatic (V0.32). Filtered high-quality data were compared with the reference genome (HISAT2 v2.1.0) in the Ensembl database, and exomePeak (v2.13.2) used to identify peaks in each sample and differentially methylated peaks in compared samples. Peaks were annotated according to Ensembl database annotation information, peaks in different regions [5’ untranslated region (5’UTR), coding sequences (CDS), and 3’ untranslated region (3’UTR)] of each transcript counted in every sample, and the resulting data used for motif analysis with MEME-ChIP software.

### mRNA-Seq

After five 24-week-old mice in each group were euthanized, total RNA samples were harvested from heart tissue specimen. RNA concentrations were determined using a NanoDrop ND-1000, and total RNA samples enriched using oligo dT (rRNA removal) and then selected using a KAPA Stranded RNA-Seq Library Prep Kit (Illumina) for library construction. Constructed libraries were quality assessed using an Agilent 2100 Bioanalyzer, and quantified by qPCR. Mixed libraries containing different samples were sequenced using the Illumina NovaSeq 6000 sequencer. Solexa pipeline version 1.8 (Off-Line Base Caller software, version 1.8) software was used for image processing and base identification. FastQC (v0.11.7) software was applied to evaluate sequencing read quality after adapter removal, Hisat2 (v2.1.0) software for comparison with the reference genome, and StringTie (v1.3.3) software to estimate transcript abundance, with reference to official database annotation information. The Ballgown package in R software was applied to calculate fragments per kilobase of transcript per million mapped reads at the gene and transcript levels, and screen out genes differentially expressed between samples or groups.

### MeRIP-qPCR Validation

Five genes with differentially methylated sites according to MeRIP-seq were tested by reverse transcription (RT) qPCR. After total RNA samples were harvested from heart tissue specimen, total RNA were carried out mRNA-specific enrichment and fragmentation through Arraystar Seq-Star TM poly(A) mRNA Isolation Kit (AS-MB-006-01/02). Then the interrupted mRNA fragments were enriched by m^6^A antibody [Affinity purified anti-m^6^A rabbit polyclonal antibody (Synaptic Systems, 202003)] and IgG antibody [Dynabeads^TM^ M-280 Sheep Anti-Rabbit IgG (Invitrogen, 11203D)]. Next, we eluted the RNA bound by m^6^A antibody and IgG antibody, and reverse transcribed into cDNA with random primers. RT-qPCR was performed on the input control and m^6^A-IP-enriched samples using gene-specific primers (Table 1).

**TABLE 1:** The gene-specific qPCR primers used were as follows.

Gene	Forward and reverse primer
MEF2A	F:5′ ACACCCTTAATGAATTGATGAC 3′
	R:5′ CAACATACAGCTTTGGCTTATA 3′
KLF15	F:5′ CATCCTCCAACTTGAACCTGC 3′
	R:5′ GAGGTGGCTGCTCTTGGTGT 3′
BCL2L2	F:5′ GGTCCTAAGAGCTGCCATCC 3′
	R:5′ TCAGCCACTAGAGCCCGTGT 3′
CD36	F:5′ CTTTAGGAGAAGAAATGGTGGT 3′
	R:5′ AATCTTTTGAAATATGCTGTGACT 3′
SLC25A33	F:5′ GAAACAGCGAAGGCCATAGAA 3′
	R:5′ AGCCCAGTAAGCACAAAGGAG 3′

### Statistical Analysis

All data are from three or more independent experiments and are presented as mean ± standard deviation. Statistical analyses were conducted GraphPad Prism 5.0 software. Comparisons of DCM and normal control (NC) samples were conducted using the paired Student’s *t-*test. Differences among three or more groups were assessed by one-way analysis of variance (ANOVA). Differences with *P* < 0.05 were defined as statistically significant.

## Results

### Cardiac Dysfunction and Myocardial Fibrosis in DCM Are Linked to Changes in Global m^6^A Levels

**FIGURE 1:**
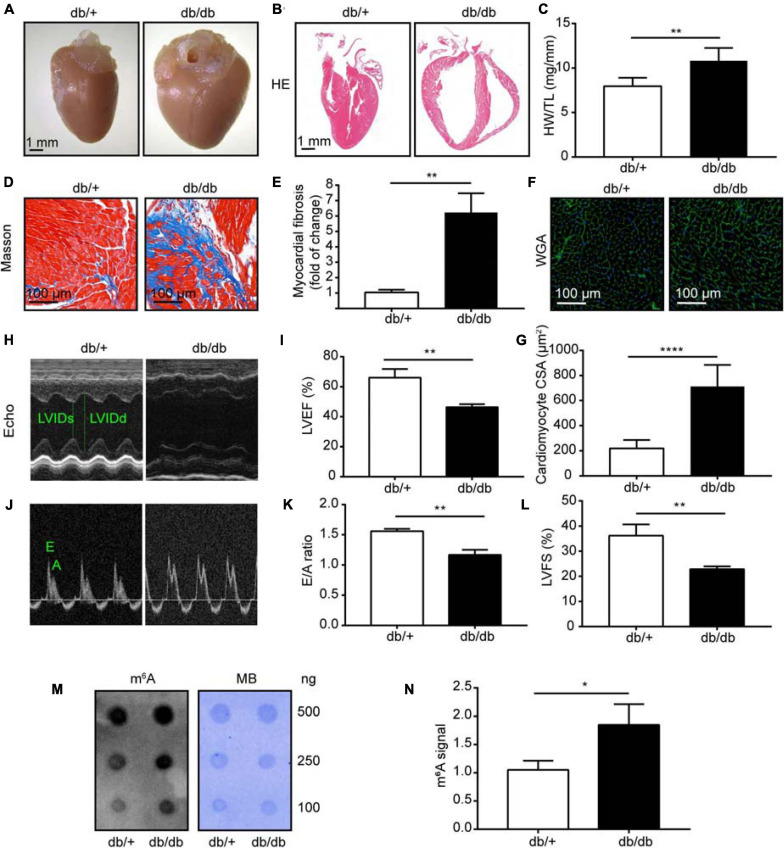
Heart morphology and function were significantly changed in db/db (DCM) mice compared with those in normal control (NC) mice. **(A)** Representative images of hearts from DCM and NC mice. **(B)** Gross morphology of mouse hearts stained with hematoxylin and eosin (HE); scale bar, 1 mm. **(C)** Ratio of heart weight to tibia length. **(D,E)** Representative images of Masson trichrome stained (Masson) hearts and quantitative analysis of interstitial fibrosis; scale bar, 100 μm. **(F,G)** Representative images of wheat germ agglutinin staining (WGA) and quantitative analysis of cardiomyocyte cross-sectional area; scale bar, 100 μm. **(H)** Representative M-mode echocardiography images; left ventricular internal diameter in systole (LVIDs) and left ventricular internal diameter in diastole (LVIDd) are labeled. **(I)** LVEF, left ventricular ejection fraction. **(J,K)** Representative Doppler echocardiography images and E/A ratio. **(L)** LVFS, left ventricular fractional shortening. **(M)** Representative dot blot showing m^6^A levels in hearts from DCM and NC mice; MB, methylene blue staining. **(N)** Quantification of m^6^A levels in hearts from DCM and NC mice. DCM, diabetic cardiomyopathy group; NC, normal control group; *n* = 5. Data are presented as mean ± SEM; differences between two groups were analyzed using the Student’s *t*-test; ****P < 0.0001, **P < 0.01, *P < 0.05 vs. NC.

Leptin receptor deficient db/db mice are mature animal models of type 2 diabetes mellitus and diabetic cardiomyopathy (DCM). Cardiac hypertrophy and fibrosis are pervasive characteristics in DCM, and are usually present in mice with DCM at the age of 24 weeks (Tan et al., 2020). In our study, db/db mice manifested obvious cardiac hypertrophy, with significantly enlarged hearts, compared with db/ + mice (Figures 1A,B), and also had evidently elevated heart weight to tibia length ratios (*n* = 5, *P* < 0.001; Figure 1C). Further, db/db mice had clearly increased interstitial fibrosis (*n* = 5, *P* < 0.001; Figures 1D,E), as well as markedly elevated cardiomyocyte cross-sectional area (*n* = 5, *P* < 0.0001; Figures 1F,G).

To provide further evidence that cardiac function was maladjusted, we performed serial echocardiography in NC and DCM group mice at 24 weeks old. The db/db mice manifested decreased cardiac function at 24 weeks old, and significantly reduced left ventricle ejection fraction (LVEF) and left ventricle fractional shortening (LVFS) compared with NC mice (*n* = 5, *P* < 0.0001; Figures 1H,I,L). Furthermore, mice with DCM showed a significantly decreased E to A wave ratio (E/A) compared with the NC group (*n* = 5, *P* < 0.0001; Figures 1J,K), indicating that the diastolic function of the heart was abnormal in DCM mice. Blood glucose (BG), triglyceride (TG), and total cholesterol (TC) were also significantly increased in db/db (DCM) mice at 16 weeks compared with db/ + mice (Supplementary Figure 1), indicating that db/db mice had abnormal blood glucose and lipid metabolism. Overall, these data demonstrate that we successfully constructed a mouse model of DCM.

Next, to assess global m^6^A levels in db/db and db/ + mouse hearts, we performed dot blot analysis on heart samples from both groups and found relatively higher total m^6^A levels in db/db mice than the db/ + mice (Figures 1M,N; *P* < 0.05). Therefore, we conducted transcriptome-wide MeRIP-seq and RNA-Seq to generate an m^6^A-methylation map of DCM.

### Transcriptome-Wide MeRIP-Seq Demonstrates Differential m^6^A Modification Patterns in DCM Compared With NC Mouse Hearts

**FIGURE 2:**
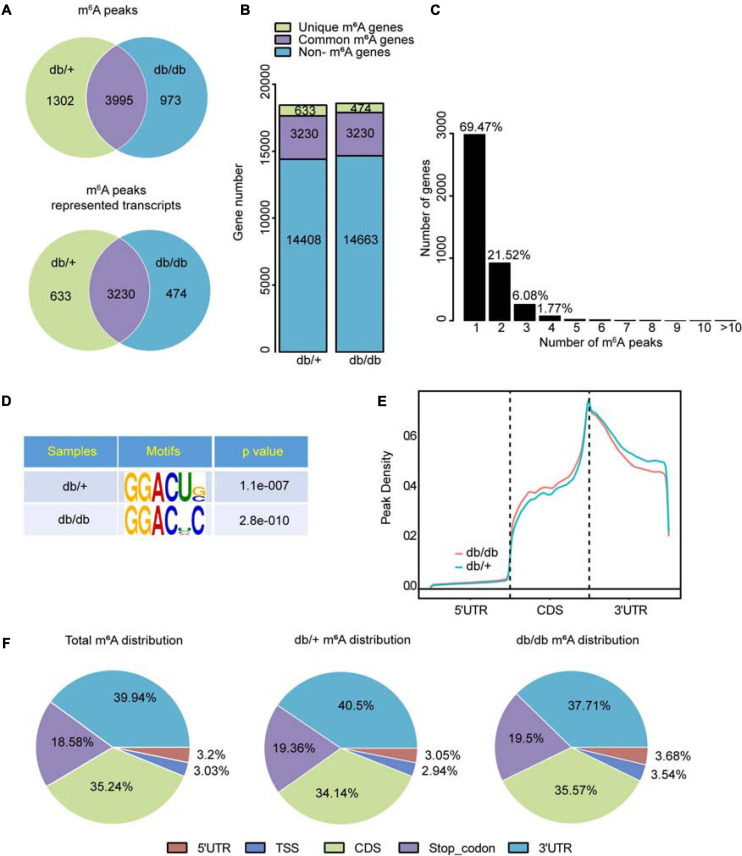
Transcriptome-wide m^6^A-seq and analysis of m^6^A peaks. **(A)** Overlap of m^6^A peaks in db/ + and db/db samples. Identification of m^6^A peaks using exomePeak; numbers of DCM-unique, control-unique, and common m^6^A peaks are shown as Venn diagram of m^6^A peaks representing transcripts in the two groups. **(B)** Summary of m^6^A-modified genes identified in m^6^A-seq. **(C)** Distribution of m^6^A-modified peaks per numbers gene. **(D)** Top m^6^A motifs enriched from all identified m^6^A peaks in the two groups. **(E)** Density curve showing the accumulation of differentially methylated m^6^A peaks in transcripts for the two groups; each transcript is divided into three regions: 5′UTR, CDS, and 3′UTR. **(F)** Proportion of m^6^A peaks distributed in the indicated regions in the NC and DCM samples; loss of existing m^6^A peaks (NC-unique peaks) or appearance of new m^6^A peaks (DCM-unique peaks) in the DCM group.

Diabetic cardiomyopathy hearts had unique m^6^A modification patterns that differed from those of NC heart samples. We identified 4968 m^6^A peaks, representing 3,704 gene transcripts, in the DCM group by model-based analysis using exomePeak (v2.13.2) (Figure 2A). In the NC group, 5297 m^6^A peaks were identified, corresponding to 3,863 gene transcripts (Figure 2A). We detected 3995 unique m^6^A peaks and 3230 m^6^A peaks associated with transcripts in both groups. The DCM heart had 973 new peaks, and 1302 new peaks were absent relative to the NC group. This finding indicates that global m^6^A modification patterns in the DCM group differed from those in the NC group (474 vs. 633; Figure 2B).

Analysis of m^6^A peak distribution showed that approximately 69.47% of modified genes had an individual m^6^A-modified peak, and the majority of genes had one to three m^6^A modification sites (Figure 2C). m^6^A methylation was further mapped using MEME-ChIP software which identified the top consensus motif in m^6^A peaks as GGACU (Figure 2D), which is similar to the previously identified RRACH motif (where *R* = G or A; *A* = m^6^A, and *H* = U, A, or C) (Dominissini et al., 2012; Meyer et al., 2012).

Within genes, m^6^A peaks were predominantly distributed in coding sequences (CDS) following the 3’ untranslated region (3’UTR) and in the immediate vicinity of the stop codon (Figure 2E). Total and unique m^6^A peaks were analyzed in DCM and NC whole transcriptome data and divided into 5’ UTR, transcription start site region (TSS), CDS, stop codon, and 3’UTR regions, based on their locations in RNA transcripts. The major regions of m^6^A peak enrichment were in CDS, 3’UTR, and stop codon vicinity regions (Figure 2F), consistent with previous m^6^A-seq results (Meyer et al., 2012). DCM-unique m^6^A peaks distributions showed a different pattern from NC-unique peaks, with a relative increase in the m^6^A residues in CDS regions and a relative decrease in 3’ UTR (35.57 vs. 34.14%; 37.71 vs. 40.5%, Figure 2F).

### Pathways Enriched for Transcripts Differentially m^6^A Methylated in DCM Are Closely Linked to Cardiac Fibrosis, Myocardial Hypertrophy, and Myocardial Energy Metabolism

Next, we identified differentially methylated transcripts and analyzed them using Gene Ontology (GO), Kyoto Encyclopedia of Genes and Genomes (KEGG) Pathway, and protein interaction network analyses.

**FIGURE 3:**
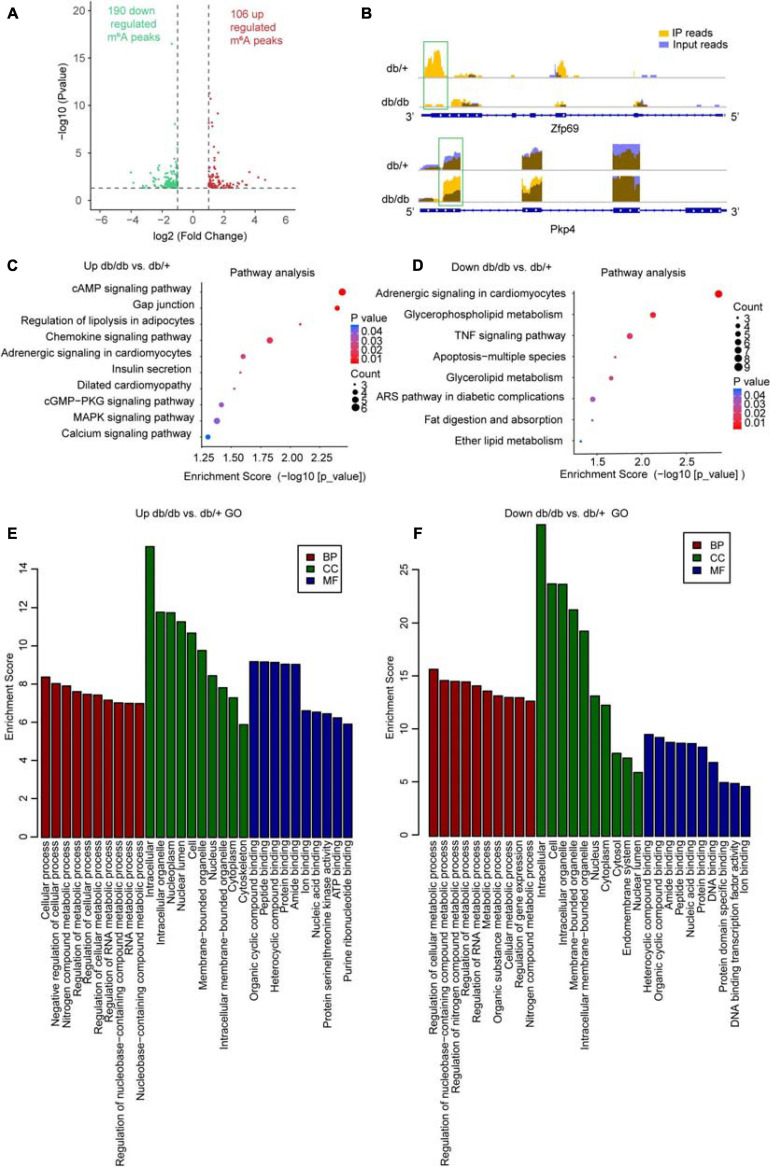
Global m^6^A modification changes in DCM hearts compared with control heart tissue. **(A)** Identification of 106 hyper-methylated and 190 hypo-methylated m^6^A peaks that were significantly increased or decreased (fold-change > 2, *P* < 0.05) in abundance, respectively, in the DCM compared with NC samples. **(B)** m^6^A abundance in *Zfp69* and *Pkp4* mRNA transcripts in the NC and DCM samples, as detected by m^6^A-seq; m^6^A peaks in green rectangles showed significantly increased or decreased abundance (fold-change > 2, *P* < 0.05) in the DCM compared with NC samples. **(C–F)** Gene ontology enrichment and pathway analyses of differentially altered m^6^A mRNA in DCM compared with NC groups; top 10 significantly enriched pathways for upregulated **(C)** and downregulated **(D)** genes; major gene ontology terms significantly enriched for up-methylated **(E)** and down-methylated **(F)** genes. ARS pathway involved in diabetic complications: AGE-RAGE signaling.

We compared the abundance of m^6^A peaks between NC and DCM samples and found among the 3995 m^6^A peaks detected in both samples, 984 differentially methylated sites were detected and selected for further study. Among them, 295 hypermethylated and 689 hypomethylated m^6^A sites were found in the DCM group [fold change (FC) > 1.5, *P* < 0.05; Figure 3A]. Differentially methylated sites in both groups showed significantly altered intensity on analysis using Integrative Genomics Viewer (IGV) software. Representative m^6^A-methylated mRNA peaks in the zinc finger domain transcription factor (*Zfp69*) and plakophilin-4 (*Pkp4*) genes are shown in Figure 3B as examples of sites with decreased and increased m^6^A levels, respectively.

To determine the potential biological significance of changes in m^6^A methylation associated with DCM, we conducted GO analysis of differentially methylated RNAs. The results revealed that, compared with db/ + mice, hypermethylated and hypomethylated RNAs in db/db mice were particularly associated with metabolism-related terms; for example, regulation of metabolic process, RNA metabolic process, organic substance metabolic process, cellular metabolic process, regulation of gene expression, nucleobase-containing compound, and nitrogen compound metabolic process, indicating that the differentially methylated RNAs were closely associated with metabolism (Figures 3E,F). Further, KEGG Pathway analysis of RNAs differentially methylated in DCM were mainly associated with cardiac fibrosis, myocardial hypertrophy, and myocardial energy metabolism; for example, the cAMP signaling pathway, dilated cardiomyopathy, and cGMP-PKG signaling pathway, which are strongly associated with myocardial hypertrophy (Figures 3C,D). The chemokine signaling pathway, adrenergic signaling in cardiomyocytes, TNF signaling pathway, and the advanced glycation end products (AGEs)- receptors for AGEs (RAGE) pathway (which is involved in diabetic complications) also appear to be important mechanisms associated with cardiac fibrosis (Figures 3C,D). Further, glycerophospholipid metabolism, apoptosis-multiple species, glycerolipid metabolism, fat digestion and absorption, ether lipid metabolism, and the calcium signaling pathway associated with myocardial energy metabolism were enriched among differentially methylated transcripts (Figures 3C,D). Overall, our data indicate that these differentially methylated RNAs may be involved in DCM pathogenesis.

Protein interaction network analysis of genes with differentially methylated transcripts was performed using Cytoscape software. BCL2L2, MEF2A, and VEGF-A were the most central proteins, and are particularly associated with the advanced glycation end products (AGEs)-RAGE pathway, which is involved in diabetic complications (Supplementary Figure 2). Therefore, these genes and the corresponding signaling pathways are likely of great importance in protein-protein interaction networks and molecular events underlying DCM.

In summary, transcripts with DCM-unique m^6^A peaks were are closely related to cardiac fibrosis, myocardial hypertrophy, and myocardial energy metabolism, which are major pathological features of left ventricle remodeling in DCM.

### Combined Analysis of MeRIP-seq and RNA-Seq Data Reveals That Unique m^6^A-Modified Transcripts Were Highly Relevant to Left Ventricle Remodeling Pathological Features of DCM

**FIGURE 4:**
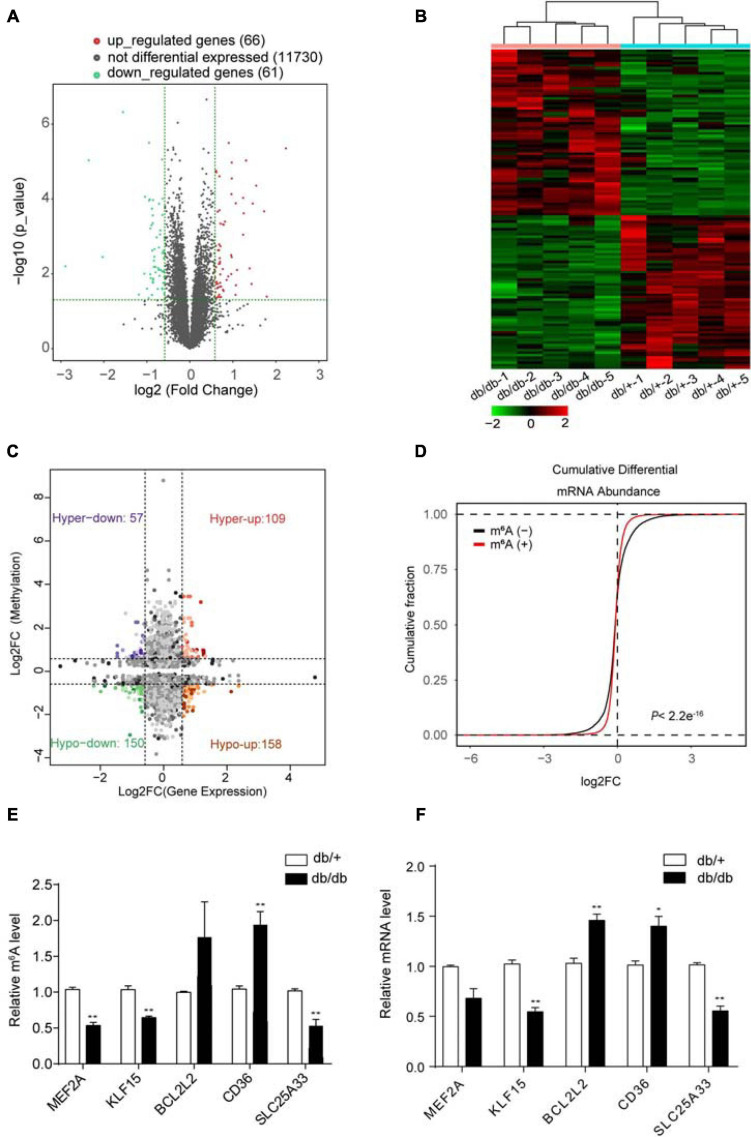
Combined analysis of RNA-seq and MeRIP data comparing DCM and NC samples. **(A)** Volcano plots showing mRNAs significantly differentially expressed in comparisons between DCM and NC samples (fold-change > 1.5 and *P* < 0.05). **(B)** Cardiac clustering analysis of differentially expressed mRNAs (*P* < 0.01). **(C)** Four quadrant graph showing the distribution of transcripts with significant changes in m^6^A-modification level and corresponding mRNA expression (fold-change > 1.2 and *P* < 0.05). **(D)** Cumulative distribution of mRNA expression changes between DCM and NC samples for m^6^A-modified genes (red) and non-target genes (black). *P* values were calculated by two-sample Kolmogorov-Smirnov test. **(E)** MeRIP-qPCR validation of m^6^A level changes in five representative hyper-methylated or hypo-methylated genes in NC and DCM group samples. **(F)** Relative mRNA levels of five representative genes were measured by real-time PCR in normal control and DCM group samples. Data are expressed as mean ± SD; data were analyzed using the Student’s *t*-test. *P < 0.05; **P < 0.01 vs. NC group (*n* = 3 per group).

RNA-Seq data showed that 127 mRNAs were significantly dysregulated in DCM samples compared with NCs, including 61 downregulated and 66 upregulated mRNAs (FC > 1.5, *P* < 0.05; Figure 4A). Hierarchical clustering analysis of RNA-Seq data showed that the trend in differential gene expression between the groups was consistent among individual samples within each group (*n* = 5 per group) (Figure 4B). Further, principal component analysis showed that samples from the DCM and NC groups clustered separately, with only small differences among samples within each group (Supplementary Figure 3). Interestingly, GO and KEGG pathway analyses showed that differentially expressed genes were mainly associated with cardiac fibrosis, myocardial hypertrophy, and myocardial energy metabolism (Supplementary Figures 4A,B, 5A,B), consistent with involvement in myocardial remodeling pathology (Ritchie and Abel, 2020).

Next, we performed combined analysis of MeRIP-seq and RNA-Seq data to identify target genes that were modified by m^6^A. We detected 166 hypermethylated m^6^A peaks within mRNA transcripts, 57 and 109 of which were significantly downregulated and upregulated, respectively (Figure 4C). Further, 308 hypomethylated m^6^A peaks were detected in mRNA transcripts, with 158 and 150 clearly upregulated and downregulated, respectively (Figure 4C). The top three ranking genes showing the most distinct changes in m^6^A and mRNA levels in DCM samples relative to NC in each quadrant of Figure 4C are presented in Table 2. Comparisons of the whole transcriptome of DCM versus NC, m^6^A methylated genes were more upregulated than downregulated in DCM. This trends did not exist for non-m^6^A methylated genes (Figure 4D).

**TABLE 2:** The ranking of the top 3 genes in each quadrant graph.

		m^6^A level change					mRNA level change		
Pattern	Chromosome	Peak region	Peak start	Peak end	Fold Change	*P* value	Strand	Fold Change	*P* value
Hyper-up	chr6	cds	140623718	140633781	1.97794	1.31826E-09	+	1.880101649	0.021856265
Hyper-up	chrX	cds, utr5, TSS	20688477	20693415	1.69349	0.000144544	+	2.40714967	0.015432908
Hyper-up	chr17	utr5, cds, TSS	28438026	28441603	1.99308	0.000020893	−	2.070126156	0.0063377
Hyper-down	chr1	cds	131977633	131977962	1.94531	0.0147911	+	0.60843951	0.04150425
Hyper-down	chr7	cds	44516146	44517004	1.68646	0.00812831	−	0.550175197	0.000444612
Hyper-down	chr2	cds	181501928	181508184	1.63354	2.75423E-05	+	0.504849429	0.014587297
Hypo-up	chr8	utr3	66468623	66468863	0.34151	0.0281838	+	1.572498891	0.031693695
Hypo-up	chr10	utr3	88473835	88474016	0.441351	9.12011E-09	−	1.627484569	0.019479321
Hypo-up	chr9	utr3	113919351	113919682	0.665265	0.000501187	+	2.101866893	0.020272987
Hypo-down	chr17	cds	35852716	35852926	0.395021	0.0190546	+	0.625425926	0.009485677
Hypo-down	chr4	utr3	149744394	149744665	0.609205	0.0040738	−	0.523997734	0.00010282
Hypo-down	chr5	utr3	16374390	16374511	0.577143	0.000331131	+	0.651881805	0.016484324

Furthermore, combined analysis of MeRIP-seq and RNA-Seq data demonstrated that unique m^6^A-modified transcripts were highly relevant to cardiac fibrosis, myocardial hypertrophy, and myocardial energy metabolism; therefore, we focused on genes critical for these processes, including *Mef2a*, *Klf15*, *Bcl2l2*, *Cd36*, and *Slc25a33* (Figure 4C and Table 2).

We used quantitative reverse-transcription PCR (RT-PCR) to validate the key genes *Mef2a*, *Klf15*, *Bcl2l2*, *Cd36*, and *Slc25a33*, which are associated with DCM pathophysiology and found that all of them were significantly enriched in immunoprecipitation (IP) pull-down samples (Figure 4E). Further, the mRNA levels of these genes were measured in db/ + and db/db hearts samples (Figure 4F), and the results showed that they all had similar mRNA expression tendencies consistent with their m^6^A-methylation levels (Figure 4F).

In summary, these data suggest that differentially methylated RNAs affect cardiac fibrosis, myocardial hypertrophy, and myocardial energy metabolism, thereby affecting protein homeostasis in a transcription-independent manner.

### FTO Is Downregulated in DCM, and Overexpression of FTO Improves Cardiac Function by Reducing Myocardial Fibrosis and Myocyte Hypertrophy

**FIGURE 5:**
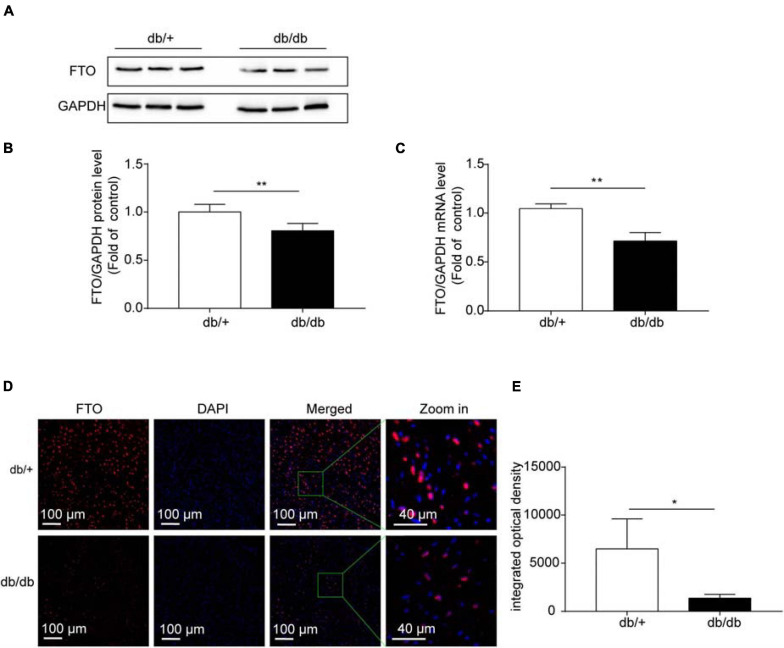
Fat mass and obesity-associated (FTO) protein levels were significantly downregulated in diabetic cardiomyopathy. **(A,B)** Representative western blots and quantitative analysis of FTO/GAPDH protein expression in hearts from DCM and NC mice (*n* = 6 per group). **(C)** *Fto* mRNA levels in hearts from DCM and NC mice (*n* = 6 per group). **(D)** Confocal immunofluorescence using specific antibodies against FTO (red) in DCM hearts. Nuclei were stained with DAPI (blue), merged images show co-localization. Scale bar, 100 μm. **(E)** Quantification of FTO fluorescence intensity. Data are presented as mean ± SEM; differences between two groups were analyzed using the Student’s *t*-test; *P < 0.05 vs. NC, **P < 0.01 vs. NC.

Based on the results of GO/KEGG analysis of unique genes and combined analysis of MeRIP-seq and RNA-Seq data in DCM and NC hearts, we suspected that the demethylase, FTO, may have an important role in DCM pathogenesis, which is closely related to energy metabolism regulation. Therefore, in the next experiment, we assessed the role of FTO in the mechanism underlying DCM. FTO was downregulated in db/db mice compared with db/ + group. The mRNA and protein expression levels of the FTO demethylase enzyme were measured in hearts from the db/db and db/ + groups to determine whether fibrosis is related to m^6^A modification. FTO protein levels estimated by western blotting were significantly decreased in the db/db group compared with db/ + group (*P* < 0.001; *n* = 6/group; Figures 5A,B). *Fto* mRNA expression was also significantly decreased in db/db mice compared with db/ + controls (*P* < 0.001; *n* = 6/group; Figure 5C). In addition, immunostaining showed that FTO was predominantly expressed in cell nuclei, and that integrated optical density of FTO was decreased in db/db mice compared with the db/ + group (*P* < 0.05; *n* = 6/group; Figures 5D,E). We also detected expression of other important methyltransferases and demethylases, including Mettl3, Mettl14, and ALKBH5; however, no significant differences were detected (Supplementary Figure 6).

**FIGURE 6:**
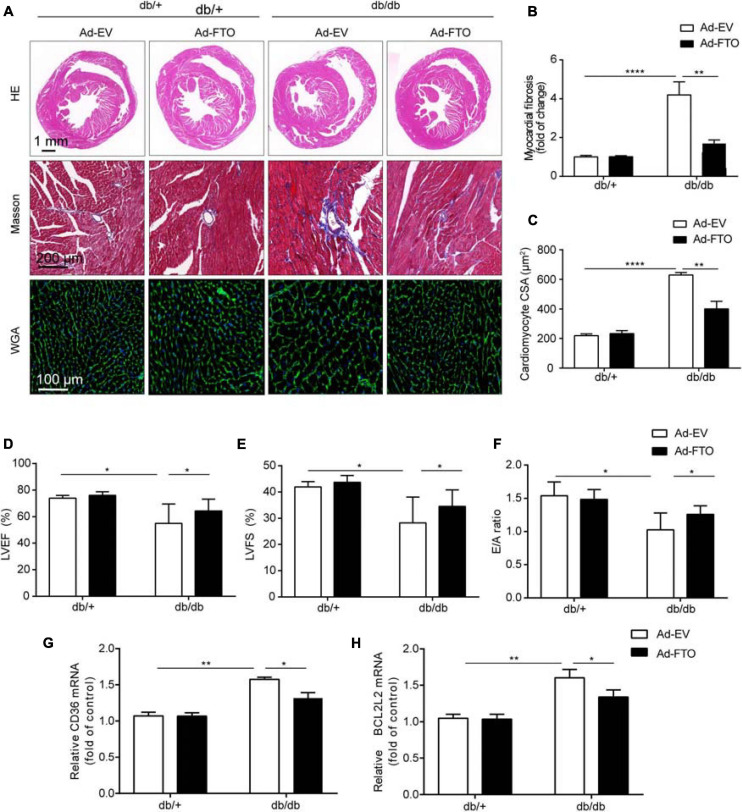
Reconstitution of FTO alleviated cardiac hypertrophy and fibrosis in diabetic db/db mice. **(A)** The gross morphology of hearts stained with HE, Masson, and WGA. Scale bar, 2 mm **(B,C)** Quantitative analysis of interstitial fibrosis and cardiomyocyte cross-sectional area. Scale bar, 20 μm. Mice with reconstituted FTO showed significantly increased ejection fraction **(D)**, fractional shortening **(E)**, and E/A ratio **(F)**. Mice with reconstituted FTO showed reduced *Cd36* **(G)** and *Bcl2l2* **(H)** expression, as determined by qPCR. Data are expressed as mean ± SD and were analyzed using the Student’s *t*-test. *P < 0.05; **P < 0.01, ****P < 0.0001 vs. NC group (*n* = 3 per group).

Overexpression of FTO significantly improved cardiac function by reducing myocardial fibrosis and myocyte hypertrophy in db/db mice. To determine whether overexpression of FTO protected hearts against DCM, adeno-associated virus vectors encoding FTO were injected into 16-week-old db/db or db/ + mice via a tail vein and FTO expression assessed 8 weeks later (Supplementary Figure 7A). Myocardial FTO expression was increased approximately 2.2-fold in db/db mouse hearts 8 weeks after injection of FTO-expressing adeno-associated virus (AAV-FTO) (Supplementary Figures 7B,C). Overexpression of FTO leaded to decrease total m^6^A levels in db/db mice (Supplementary Figures 7D,E); *P* < 0.05). Eight weeks after adenovirus injection, reconstitution of FTO efficiently prevented myocardial fibrosis by reducing interstitial fibrosis in db/db mouse hearts (Figures 6A,B). Further, reconstitution of FTO efficiently reduced myocyte hypertrophy, as evidenced by decreased cardiomyocyte cross-sectional area in db/db mouse hearts (Figures 6A,C). Moreover, reconstitution of FTO significantly enhanced cardiac function in db/db mice by increasing LVEF and LVFS (Figures 6D,E). Furthermore, Doppler echocardiography indicated that FTO reconstitution also alleviated diastolic dysfunction in db/db mice by elevating the E/A ratio (Figure 6F). Overexpression of FTO decreased the mRNA levels of *Bcl2l2* and *Cd36* (Figures 6G,H). Taken together, these data indicate that reconstitution of FTO prevented myocardial fibrosis and myocyte hypertrophy, and overexpression of FTO improved cardiac systolic and diastolic function in db/db mice.

## Discussion

Diabetic cardiomyopathy is a specific type of cardiomyopathy caused by abnormal metabolism during diabetes. The main features of DCM are myocardial hypertrophy, cardiac fibrosis, coronary microvascular dysfunction, left ventricular enlargement, and weakened ventricular wall motion (Ritchie and Abel, 2020; Tan et al., 2020). In the early stage of DCM, echocardiography mainly indicates diastolic dysfunction, while later stage disease is manifested as abnormal systolic function (Tan et al., 2020). Although multiple aspects of epigenetic regulation, from DNA to protein modification, have been extensively studied in DCM, the role of RNA modification in the regulation of gene expression is just beginning to be explored. Previous studies have demonstrated that m^6^A dysregulation is associated with cardiac homeostasis and diseases, such as cardiac hypertrophy, cardiac remodeling, myocardial infarction, and heart failure (Dorn et al., 2019; Mathiyalagan et al., 2019; Mo et al., 2019; Song et al., 2019; Berulava et al., 2020; Gao et al., 2020; Lin et al., 2020); however, the transcriptome-wide distribution of m^6^A in DCM remains largely unknown. Our study reveals that m^6^A RNA methylation is altered in db/db mice, which exhibit unique m^6^A modification patterns that differ from those in db/ + mice at the transcriptome-wide and gene-specific scales. GO and KEGG analyses revealed that genes with m^6^A RNA methylation differences between db/db and db/ + mice were particularly associated with cardiac fibrosis, myocardial hypertrophy, and myocardial energy metabolism. FTO was downregulated in db/db mice compared with db/ + mice, and overexpression of FTO in db/db mice improved cardiac function and significantly reduced myocardial fibrosis and myocyte hypertrophy. FTO is a critical RNA-modifying enzyme that may control cardiomyocyte function by catalyzing the demethylation of m^6^A on specific subsets of mRNAs; for example, *Mef2a*, *Klf15*, *Bcl2l2*, *Cd36*, and *Slc25a33*.

The m^6^A modification patterns in db/db mice differed from those of db/ + mice at both the transcriptome-wide and gene-specific scales. We detected 4,968 m^6^A peaks in DCM, consistent with the 3,208 and 3,922 peaks described in heart failure and myocardial hypertrophy, respectively (Mathiyalagan et al., 2019; Berulava et al., 2020). Together, these results indicate that m^6^A is a ubiquitous post-transcriptional RNA modification in cardiovascular diseases. Furthermore, we investigated differentially methylated RNAs during DCM development and found that numbers were higher than those of genes with differential mRNA levels (296 vs. 127), indicating that the changes in m^6^A RNA methylation far exceeded those in gene expression. Importantly, we found that, in DCM, m^6^A was primarily present in the CDS and 3’UTR regions, which may influence mRNA stability, translation efficiency, and alternative splicing. Therefore, we speculate that the differential methylation of RNA in DCM may influence RNA at the post-transcription and translation levels, and particularly translation efficiency.

Gene Ontology and Kyoto Encyclopedia of Genes and Genomes analyses showed that transcripts with differential m^6^A methylation in DCM were significantly enriched in processes and pathways associated with myocardial energy metabolism, such as glycerophospholipid metabolism, glycerolipid metabolism, and regulation of cellular metabolic processes (Figures 4C–F). DCM is defined as a loss of flexibility in myocardial substrate metabolism, which leads to mitochondrial dysfunction, inflammation, and myocardial fibrosis (Peterson and Gropler, 2020). Cardiomyocyte ATP is mainly (60–90%) derived from fatty acid fat β oxidation under physiological conditions (Peterson and Gropler, 2020); however, in the diabetic state, fatty acid oxidation can produce numerous lipid intermediates, which accumulate in cardiomyocytes to cause lipotoxicity and ultimately lead to impaired heart function. Further, excessive fatty acid oxidation can cause accumulation of reactive oxygen species (ROS), leading to oxidative stress, which damages myocardial cells (Peterson and Gropler, 2020; Ritchie and Abel, 2020). We propose that m^6^A methylation may be involved in the key pathogenic processes underlying DCM, including abnormal myocardial substrate metabolism. Furthermore, our RNA-Seq data demonstrate that abnormally up-regulated genes in the DCM samples were significantly enriched in biological processes involving lipid metabolism, cellular lipid metabolism, and fatty acid metabolism (Supplementary Figure 3A), consistent with myocardial energy metabolism pathology (Ritchie and Abel, 2020; Tan et al., 2020). Moreover, pathway analysis showed that unsaturated fatty acid biosynthesis, fatty acid elongation, butanoate metabolism, PPAR signaling pathway, and the HIF-1 signaling pathway were significantly altered among up-regulated genes (Supplementary Figure 4B). These results support that changes in m^6^A RNA methylation mainly occur in transcripts coding for proteins involved in cardiac metabolic processes, with differences in gene expression also linked to metabolic regulation (Jia et al., 2018). In addition, combined analysis of MeRIP-seq and RNA-Seq data identified the target genes *Cd36* and *Slc5a33*, which were validated by MeRIP-qPCR (Figures 4E,F). CD36 deficiency rescues lipotoxic cardiomyopathy by preventing myocardial lipid accumulation in MHC-PPARα mice (Yang et al., 2007). In our study, *Cd36* m^6^A-methylation and mRNA expression levels were upregulated, indicating that m^6^A modification may influence mRNA stability or translation efficiency.

Our GO and KEGG analyses showed that transcripts differentially m^6^A methylated in DCM were significantly enriched in processes and pathways associated with cardiac fibrosis and myocardial hypertrophy. The cAMP signaling, cGMP-PKG signaling, and dilated cardiomyopathy pathways are closely related to myocardial hypertrophy, while adrenergic signaling in cardiomyocytes, the TNF signaling pathway, the mitogen activated protein kinase (MAPK) signaling pathway, the AGEs-RAGE pathway, and chemokine signaling are strongly associated with cardiac fibrosis (Figures 4C,D). The main pathological changes in DCM include myocardial interstitial fibrosis, cardiomyocyte hypertrophy, cardiomyocyte apoptosis, and microvascular disease (Jia et al., 2018; Peterson and Gropler, 2020; Ritchie and Abel, 2020; Tan et al., 2020). In our study, GO analysis showed that metabolic processes involving nitrogen compounds were up-regulated while the cGMP-PKG signaling pathway was also increased, consistent with a previous study showing decreased NO signaling in endothelial cells and cardiomyocytes, leaded to cardiomyocyte hypertrophy by reducing the activity of soluble guanylate cyclase (sGC) and cyclic guanylate (cGMP) content, as well as cardiomyocyte loss of the protective effects of protein kinase G (PKG) in DCM (Park et al., 2018; Tan et al., 2020). Hence, our data suggest that m^6^A methylation could be involved in the important pathogenic processes underlying myocardial hypertrophy in DCM. Furthermore, AGEs promote an imbalance of inflammatory gene expression by binding to specific cell surface receptors, thus increasing matrix protein expression through the MAPK pathway in vascular and heart tissues (Jia et al., 2018). Simultaneously, AGEs are involved in increasing ROS production and promoting myocardial inflammation and fibrosis (Wang Y. et al., 2020). Our KEGG analysis of transcripts differentially m^6^A methylated in DCM showed that MAPK signaling was up-regulated, while the AGEs-RAGE pathway was down-regulated. Interestingly, enriched GO terms for genes differentially expressed in DCM based on RNA-seq data included extracellular matrix organization, myofibril assembly, and collagen-containing extracellular complex organization, indicating that m^6^A methylation may contribute to important pathogenic processes underlying cardiac fibrosis in DCM. We also validated two target genes, *Mef2a* and *Klf15*, by MeRIP-qPCR (Figures 4E,F). KLF15 affects myocardial hypertrophy by inhibition of *MEF2* and *GATA4* transcription (Zhao et al., 2019), and can reduce myocardial fibrosis by down-regulating the expression of transforming growth factor-β (TGF-β), connective tissue growth factor, and myocardial protein-associated transcription factor-A in myocardial fibroblasts (Zhao et al., 2019). Further, knockout of the *MEF2A* gene improves cardiac dysfunction and collagen deposition in DCM, while inhibition of MEF2A can reduce extracellular matrix accumulation by regulating the Akt and TGF-β1/Smad signaling pathways (Chen et al., 2015). We found significant associations between differentially m^6^A methylated transcripts and cardiac fibrosis, myocardial hypertrophy, and myocardial energy metabolism in DCM, suggesting that DCM may be regulated by epitranscriptomic processes, such as m^6^A RNA methylation.

FTO is downregulated in DCM, and overexpression of FTO improves cardiac function by reducing myocardial fibrosis and myocyte hypertrophy. The *FTO* gene was discovered in 2007 in a genome-wide association study of type 2 diabetes (Frayling et al., 2007). Further, a population cohort study found that the role of FTO risk genes is related to energy intake (Haupt et al., 2009); however, the mechanisms by which FTO influences obesity and the specific pathways related to energy metabolism remain unclear. Animal experiments showed that this increase in energy metabolism does not involve physiological exercise, and may be caused by increased activity of the sympathetic nervous system (SNS) (Church et al., 2010). Further, the increased SNS activity may promote lipolysis of fat and muscle tissues and improve fat burning efficiency, thereby reducing the occurrence of obesity (Church et al., 2010). Our KEGG pathway analysis of differentially methylated RNAs showed that they were mainly associated with adrenergic signaling in cardiomyocytes (Figure 4F). In adipogenesis, FTO can also improve the binding ability of C/EBPs with methylated or unmethylated DNA, thereby enhancing the transcription activity of the corresponding gene promoter, and stimulating preadipocyte differentiation (Wu et al., 2010). In summary, FTO plays an important role in energy metabolism.

Recent studies have shown that FTO expression is downregulated in failing mammalian hearts and hypoxic cardiomyocytes, thereby increasing m^6^A levels in RNA and decreasing cardiomyocyte contractile function (Mathiyalagan et al., 2019). Simultaneously, overexpression of FTO in mouse reduces fibrosis and promotes angiogenesis (Mathiyalagan et al., 2019). In our study, overexpression of FTO also reduced myocardial fibrosis (Figures 6A,B). In addition, FTO knockout can lead to impaired cardiac function and promote heart failure (Berulava et al., 2020). In our study, overexpression of FTO improved heart function by increasing the LVEF and LVFS (Figures 6D,E). In summary, overexpression of FTO improves cardiac function by reducing myocardial fibrosis and myocyte hypertrophy.

This study has limitations. Firstly, no human heart samples were analyzed, therefore we will further seek human heart samples to further explore the association of m^6^A with the DCM pathogenic process in the future. Second, although the target genes modified by m^6^A were detected, the mechanism by which methylation readers regulate the target genes was not explored. In the future, we will investigate whether readers influence the stability, translation efficiency, or degradation of target genes. Third, although we purposely overexpressed FTO in the heart to improve cardiac function, future experiments will use conditional knockout mice and additional DCM models to study the exact mechanism by which FTO mediates DCM.

In conclusion, m^6^A RNA methylation was altered in db/db mice, which had unique m^6^A modification patterns that differed from those in db/ + mice at the transcriptome-wide and gene-specific scales. GO and KEGG analysis indicated that differentially methylated genes were particularly associated with cardiac fibrosis, myocardial hypertrophy, and myocardial energy metabolism. FTO is downregulated in db/db mice compared with db/ + mice, and overexpression of FTO in db/db mice improved cardiac function and significantly reduced myocardial fibrosis and myocyte hypertrophy. FTO is a critical RNA-modifying enzyme that may control cardiomyocyte function by catalyzing the demethylation of m^6^A on specific subsets of mRNAs, including *Mef2a*, *Klf15*, *Bcl2l2*, *Cd36*, and *Slc25a33*.

## Data Availability Statement

The datasets presented in this study can be found in online repositories. The names of the repository/repositories and accession number(s) can be found below: GSE173384, https://www.ncbi.nlm.nih.gov/bioproject/?term=GSE173384.

## Ethics Statement

The animal study was reviewed and approved by the Ethics Committee of the Capital Institute of Pediatrics.

## Author Contributions

WJ and JW designed the experiments and wrote the manuscript. WJ, KL, and FH carried out the experiments. SO analyzed the data. JW and ZL supervised this project. All authors gave final approval for publication, and no conflict of interest exits in the submission of this manuscript.

## Conflict of Interest

The authors declare that the research was conducted in the absence of any commercial or financial relationships that could be construed as a potential conflict of interest.
