# Supplementary material for: Changes in N6-Methyladenosine Modification Modulate Diabetic Cardiomyopathy by Reducing Myocardial Fibrosis and Myocyte Hypertrophy
Source: Front Cell Dev Biol. 2021 Jul 21;9:702579. doi: 10.3389/fcell.2021.702579 (PMC8334868; doi:10.3389/fcell.2021.702579)
Supplement: Supplementary file 1 [file Data_Sheet_1.docx]

Supplementary Material

# Supplementary Figures


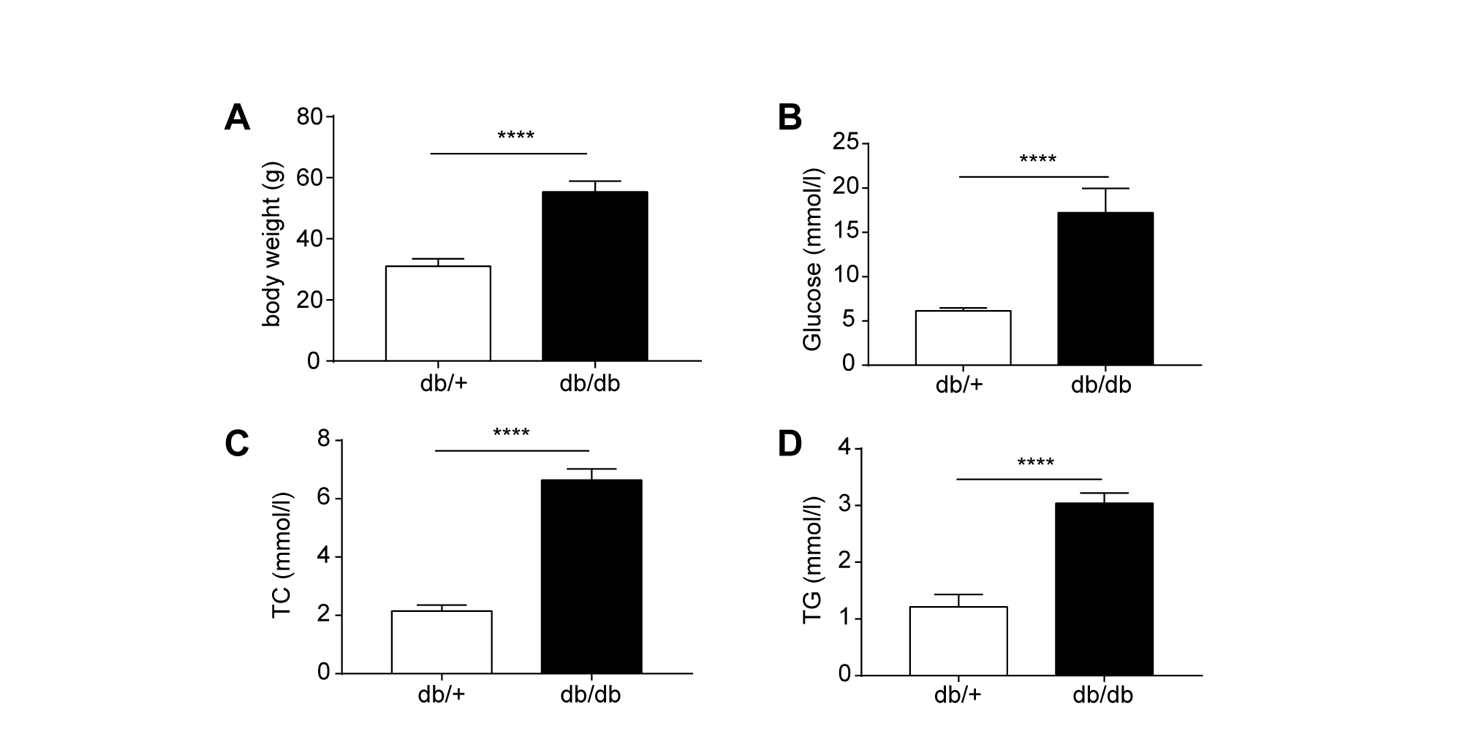


**Supplementary Figure 1.** Body weight, blood glucose, and serum lipid levels in DCM and control mice. **(A,B)** Body weight and blood glucose levels in DCM and control mice determined at 24 weeks old (n = 5 animals). **(C,D)** Serum lipids in DCM and control mice determined at 24 weeks old (n = 5 animals). *****P* < 0.0001 vs. age-matched control mice. TG, triglyceride; TC, total cholesterol.


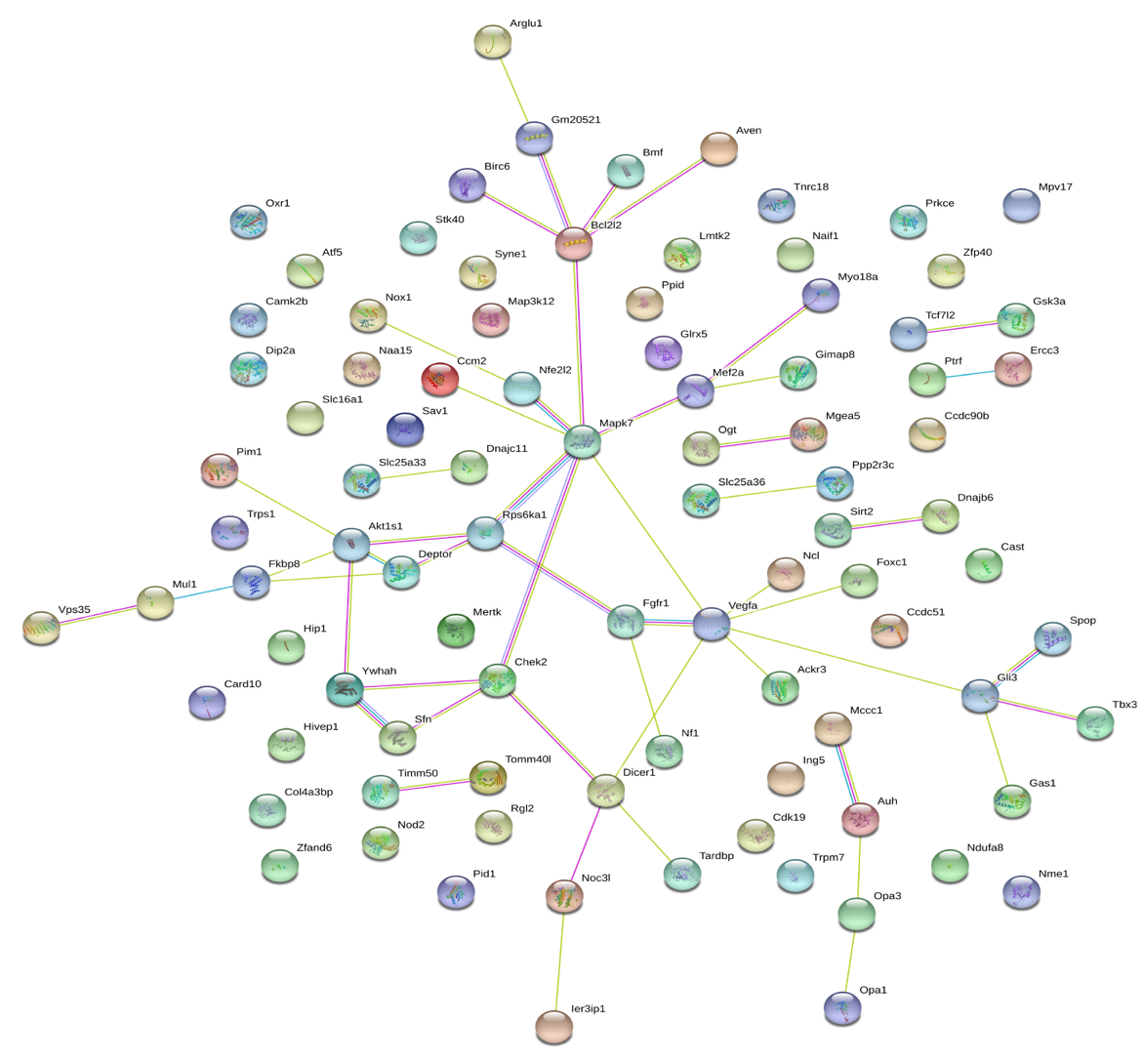


**Supplementary Figure 2.** Protein interaction network of abnormally m^6^A-modified genes. The interaction network was constructed from identified proteins, according to their m^6^A modification status in DCM relative to NC samples.


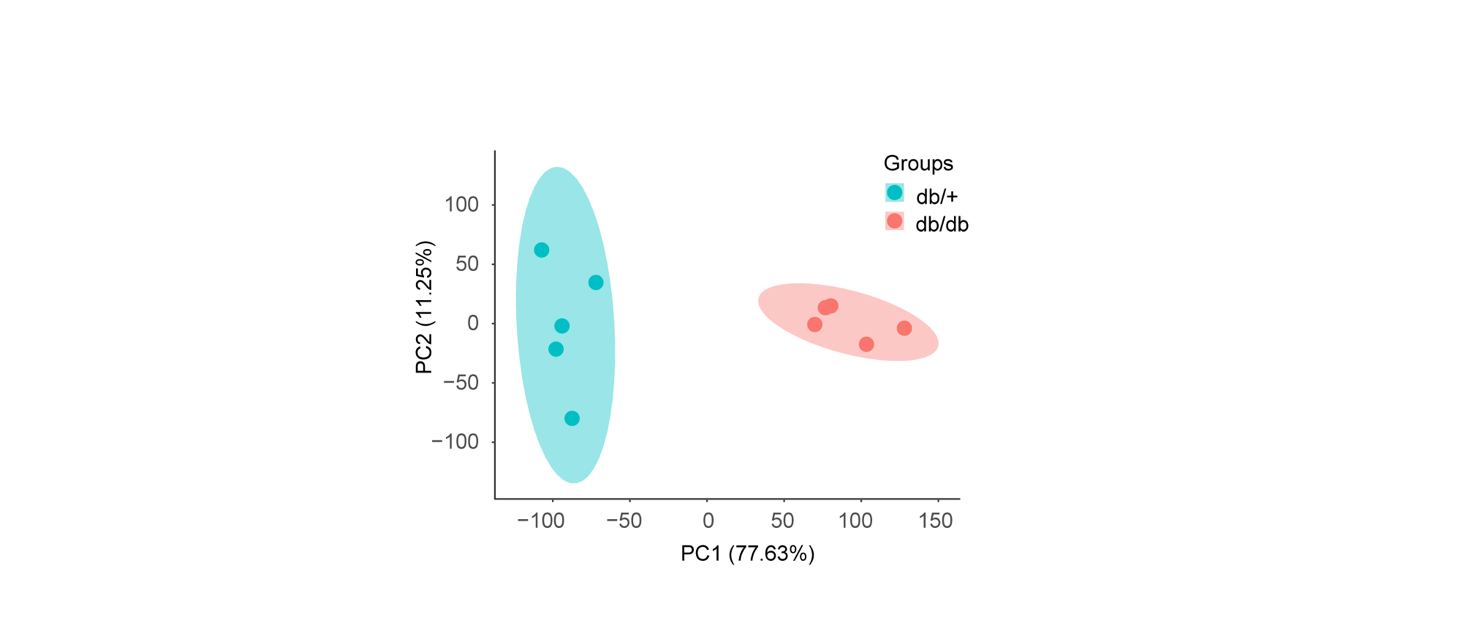


**Supplementary Figure 3.** Principle component analysis showing distinct clustering of individual samples from the DCM and NC groups. The percentages of variance associated with PC1 and PC2 were 77.63% and 11.2%, respectively.


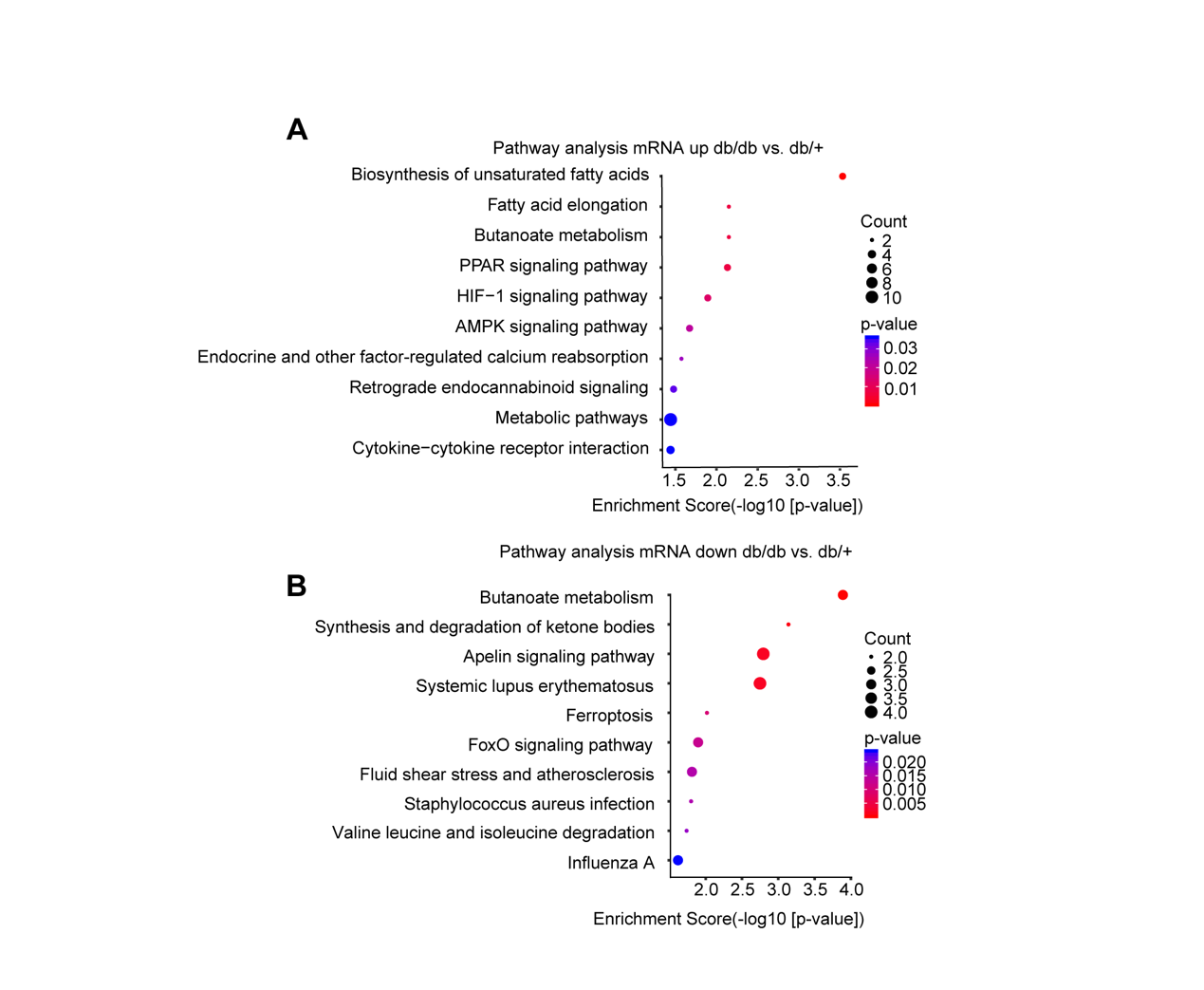


**Supplementary Figure 4.** Gene ontology enrichment of differentially expressed genes. RNA-seq. **(A)** Gene ontology analysis of upregulated genes in the NC and DCM group samples. **(B)** Gene ontology analysis of downregulated genes in the NC and DCM group samples.


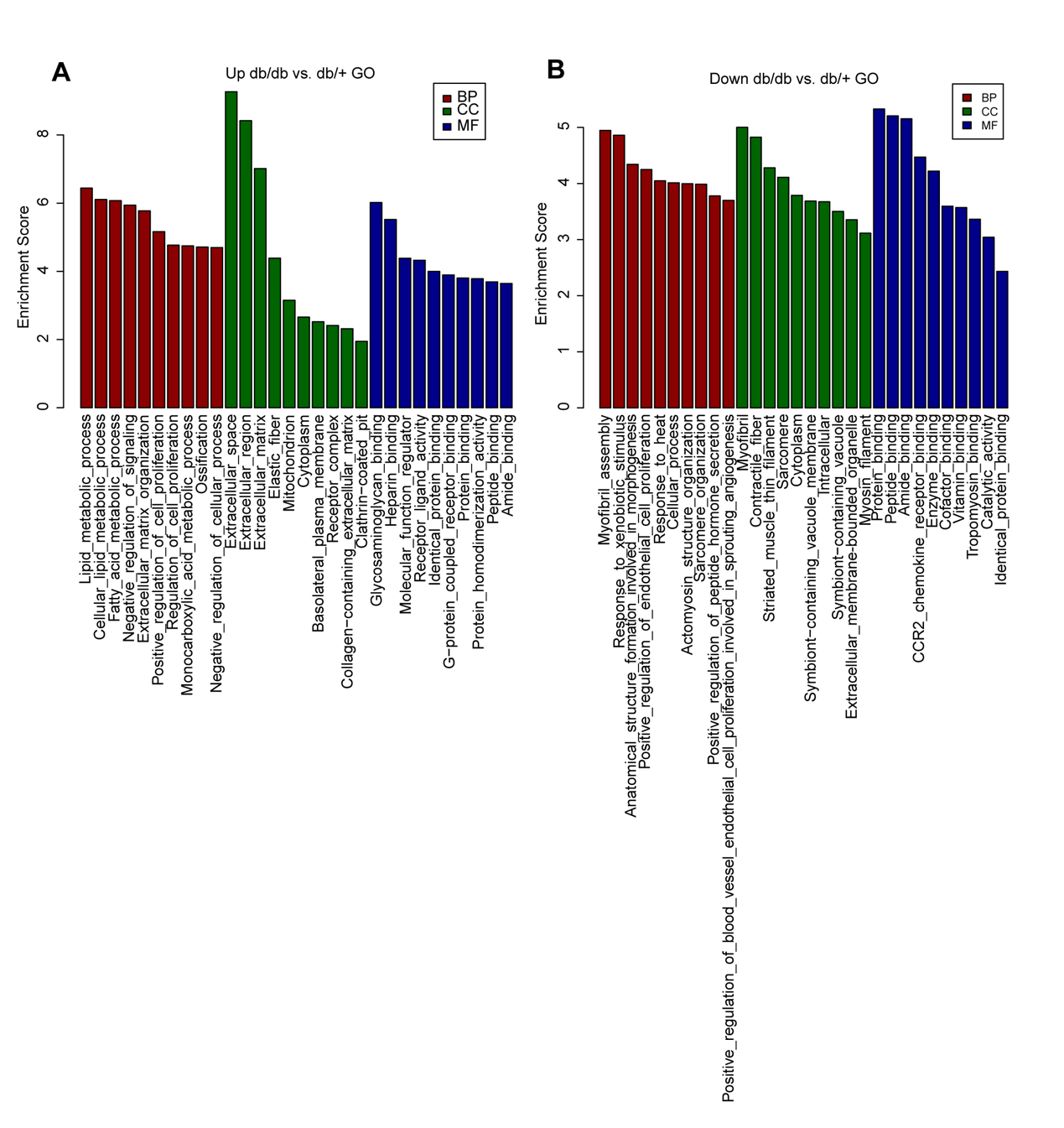


**Supplementary Figure 5.** Kyoto Encyclopedia of Genes and Genomes analysis of differentially expressed genes determined by RNA-seq. **(A)** Pathway analysis of upregulated genes in NC and DCM group samples. **(B)** Pathway analysis of downregulated genes in NC and DCM group samples


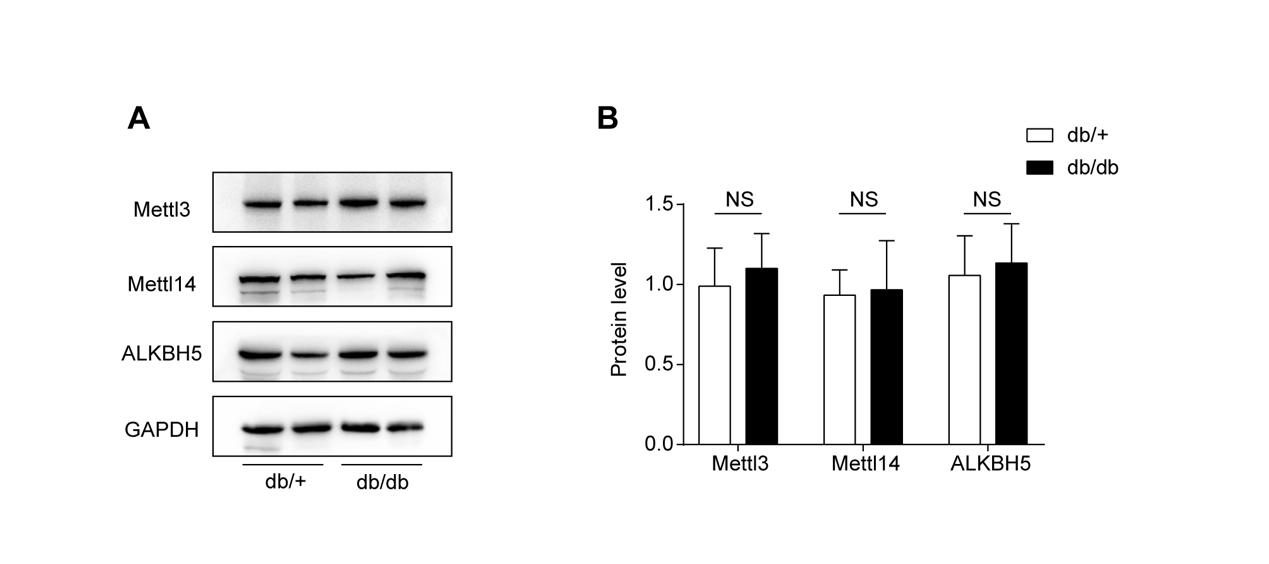


**Supplementary Figure 6.** METTL3, METTL14, and ALKBH5 levels in hearts from DCM and NC mice.

.


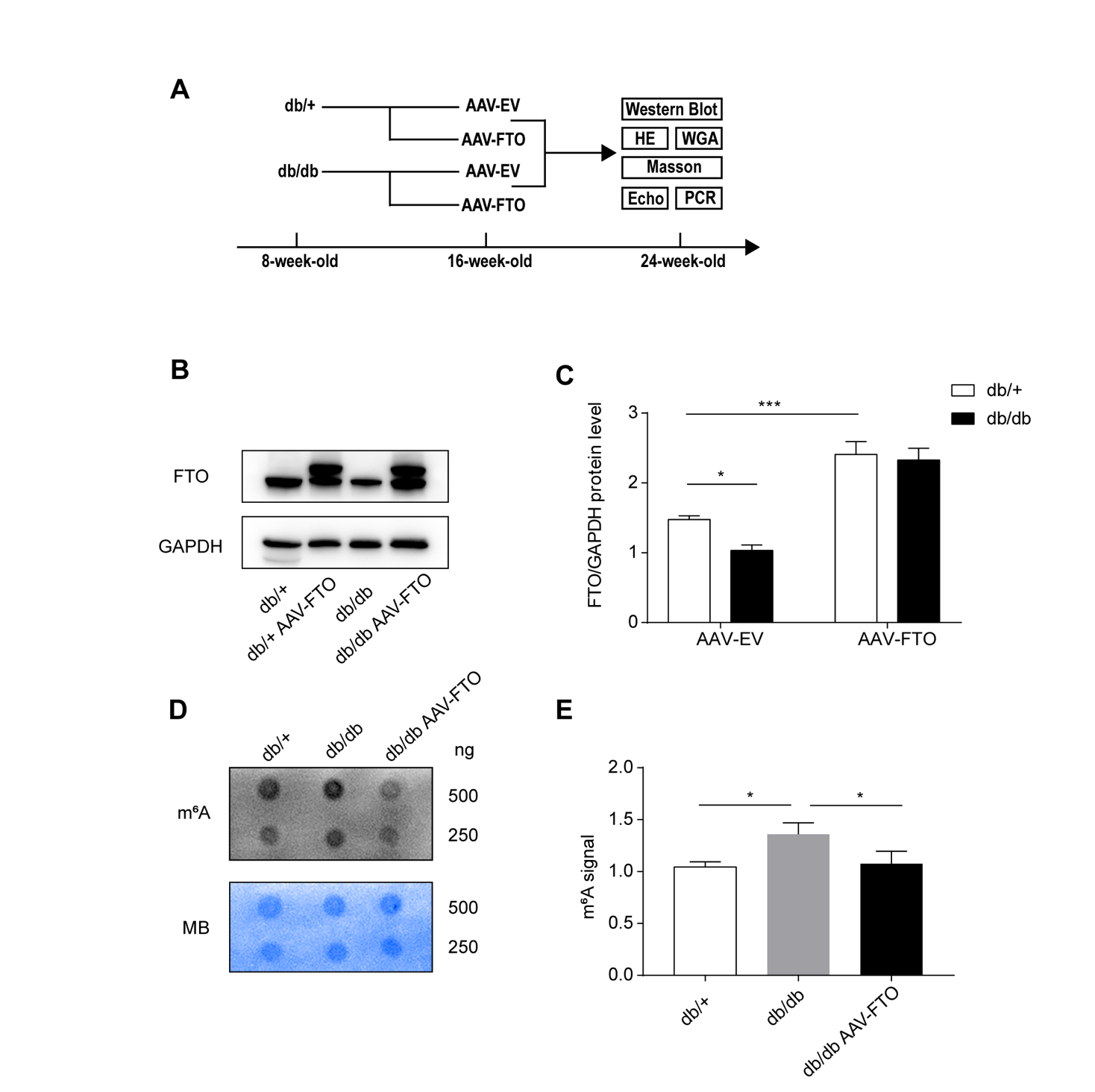


**Supplementary Figure 7.** Overexpression of FTO in db/+ and db/db mice. **(A)** Schematic representation of the experimental protocols. **(B,C)** Representative western blot images and quantitative analysis of FTO protein expression. **(D,E)** Representative dot blot images and quantitative analysis of m^6^A levels. ****P* < 0.001, **P* < 0.05
